# Unsupervised Hyperspectral Band Selection via Multimodal Evolutionary Algorithm and Subspace Decomposition

**DOI:** 10.3390/s23042129

**Published:** 2023-02-14

**Authors:** Yunpeng Wei, Huiqiang Hu, Huaxing Xu, Xiaobo Mao

**Affiliations:** School of Electrical and Information Engineering, Zhengzhou University, Zhengzhou 450001, China

**Keywords:** unsupervised band selection, multimodal evolutionary algorithm, subspace decomposition, hyperspectral image

## Abstract

Unsupervised band selection is an essential task to search for representative bands in hyperspectral dimension reduction. Most of existing studies utilize the inherent attribute of hyperspectral image (HSI) and acquire single optimal band subset while ignoring the diversity of subsets. Moreover, the ordered property in HSI is expected to be focused in order to avoid choosing redundant bands. In this paper, we proposed an unsupervised band selection method based on the multimodal evolutionary algorithm and subspace decomposition to alleviate the problems. To explore the diversity of band subsets, the multimodal evolutionary algorithm is first employed in spectral subspace decomposition to seek out multiple global or local solutions. Meanwhile, in view of ordered property, we concentrate more on increasing the difference between neighbor band subspaces. Furthermore, to utilize the obtained multiple diverse band subsets, an integrated utilization strategy is adopted to improve the predicted performance. Experimental results on three popular hyperspectral remote sensing datasets and one collected composition prediction dataset show the effectiveness of the proposed method, and the superiority over state-of-the-art methods on predicted accuracy.

## 1. Introduction

Hyperspectral imaging is an optical technology to capture consecutive spectral bands of objects. The obtained HSI is a 3-D data cube which can express the inherent properties of objects effectively. Due to the contained large amount of information, this technique has attracted considerable attentions, and been widely applied to practical field, such as remote sensing [1,2,3], chemical components analysis detection [4,5,6], and medical image analysis [7,8,9]. Despite its many successful applications, HSI itself is high-dimensional with continuous and strongly correlated band features. Such characteristic would cause information redundancy and improve computational complexity, namely “Hughes phenomenon” [10]. In this regard, an effective method is crucial for reducing dimensions and preserving data information [11].

Generally, dimension reduction of HSI can be divided into feature extraction and feature selection (also known as band selection in HSI processing) categories. For feature extraction, such as principal component analysis and manifold learning, the implementation of dimension reduction is achieved by mapping original HSI data to a lower dimensional space with a transformation matrix [12,13,14]. While for band selection, the band subset, which consists of discriminative bands, is obtained to represent original HSI information [15,16,17]. Compared with feature extraction, band selection is usually physically interpretable because it selects representative bands without changing original data information. In terms of involvement of labeled samples, band selection can be further categorized into three types: supervised [17,18,19], semi-supervised [20,21,22], and unsupervised methods [23,24,25,26]. Supervised methods evaluate obtained bands through metrics using label information, such as classification accuracy and confusion matrix. Semi-supervised band selection utilizes both labeled and unlabeled data information simultaneously. While for unsupervised band selection, it selects discriminative band subsets by the inner correlation of bands rather than label information. However, the acquisition of labeled samples is a challenge normally in many practical problems. Therefore, unsupervised band selection is necessary and mainly focused in this paper.

Unsupervised band selection can be roughly divided into four groups: greedy search, band ranking, band clustering and evolutionary algorithm (EA)-based methods [27]. A greedy search starts its band selection with an initial set, and adds or reduces bands in an iterative manner. Band ranking ranks each band with an appropriate criterion, and sorts them by their scores. High-ranked bands are selected as the expected band subset. The methods of band clustering categorize bands into different clusters. The most representative bands in each cluster are selected and constitute the desired band subsets. In EA-based methods, a set of stochastic candidate solutions is first generated. Then the genetic operators (selection, mutation, crossover) are implemented to obtain the ideal band subset during the evolutionary process. Compared with the other three methods, EA-based methods usually have a better search capability for high-dimensional problems.

Although existing studies have achieved reasonable performance, there are still two issues for unsupervised band selection. First, most of the existing methods neglect the ordered property among the spectral bands [28]. The HSI is a data cube with abundant and consecutive bands. Such structure leads to the high similarity among adjacent bands. Therefore, in view of the ordered property, selecting adjacent bands would increase the redundancy of acquired bands and deteriorate the performance of band selection. Second, most of existing studies ignore the diversity of band subsets, only select single band subset to represent HSI information. As a combination optimization problem, the optimal band subset is usually not unique, especially for high-dimensional problems [29]. It is biased to express original hyperspectral information when only using one band subset. That is, single band subset has lower generalization ability. In addition, in view of the difficulty for acquiring specific bands, it is relevant to provide multiple alternative band subsets.

To alleviate the problems of the ordered property and diversity, the subspace decomposition and multimodal optimization are potential solutions. As a special case of band clustering, subspace decomposition divides the band space into sequential subspaces rather than unordered clusters. The band subset is composed of the bands selected from each subspace. Hence, the adjacent bands will not be selected simultaneously. Multimodal optimization is a population-based and stochastic searching algorithm, which can locate multiple optimal (global or local) solutions within an independent operation. Therefore, in this paper, an unsupervised band selection method based on multimodal evolutionary algorithm and subspace decomposition (MEA-SD) is proposed. The main contributions of this paper are summarized as follows:

1. To increase the diversity of obtained solutions, the multimodal evolutionary algorithm is first applied to hyperspectral band selection. It converges the candidate solutions towards different directions. Therefore, it can seek out multiple optimal (global or local) band subsets, in which each of them can express the original HSI information well.
2. In consideration of the ordered property of spectral bands, a boundary encoding strategy and modified evaluation criterion for subspace decomposition is proposed. Different from seeking the spectral bands directly, the target of boundary encoding strategy is to find the optimal division modes of band space. Additionally, a modified evaluation criterion, endeavoring to increase the difference between neighbor subspaces rather than all clusters, is employed to evaluate the divided subspaces. Therefore, the selected bands from each subspace are scattered and lower correlative.
3. Although a single band subset can express original hyperspectral information, the generalization ability might be poor. In order to alleviate this problem, an integrated utilization strategy is employed to utilize the acquired diverse band subsets.

The remainder of this paper is organized as follows. Section 2 describes the related unsupervised band selection methods. In Section 3, the proposed band selection based on multimodal evolutionary algorithm and subspace decomposition is introduced. The details of experimental results and analysis are presented in Section 4 and Section 5. In Section 6, the advantages and limitations compared with previous studies are discussed. Finally, this paper is summarized in Section 7.

## 2. Related Work

Band selection aims to seek out the subset of most discriminative bands to represent the original HSI information. As described above, unsupervised band selection methods can be categorized into four groups: greedy-based, ranking-based, clustering-based and EA-based methods. Next, for each group, several representative methods will be introduced in this section.

### 2.1. Greedy-Based Methods

A greedy search adopts a hill-climbing method to implement band selection from an initial band subset. It iteratively adds or removes a spectral band and evaluates the current band subset with the purpose of optimizing the objective function. Band selection based on volume gradient (VGBS) is a sequential backward selection method, in which all of hyperspectral bands are regarded as a parallelotope [30]. VGBS deems that the volume of parallelotope is sensitive to redundant bands. Therefore, bands which make smaller contributions to its volume, will be removed iteratively. The remainder of the bands are considered to be discriminative. However, after each removal of bands, VGBS needs to update the gradient matrix again, which is still time-consuming. In view of this problem, a variant of VGBS (FastVGBS) is designed to reduce computational complexity further [31]. It first converts the calculation of inverse matrix of covariance matrix into recursive formula. Then, a corresponding calculation of the norm in VGBS is simplified by calculating diagonal elements of the recursive formula. Although the computational complexity is reduced to some extent, greedy-based methods still require an amount of computing time to evaluate all candidate bands. Moreover, the greedy-based methods are usually influenced by the initial selected band, and easily trapped into local optimization.

### 2.2. Ranking-Based Methods

Ranking-based methods select the discriminative band subset by sorting all bands with an appropriate criterion. The top-ranked bands are used to constitute the final band subset. Enhanced fast density-peak-based clustering (E-FDPC) is a joint band ranking and clustering method [32]. It ranks each band though weighting the local density and distance within cluster. Specifically, E-FDPC first selects the bands with top-ranked scores. The next selected band is expected to be distant from chosen bands and has a larger density in its current position. Nevertheless, how to design effective measures to evaluate density and distance within cluster is still a challenge. Band ranking based on an extended coefficient of variation (BREVC) constructs a coefficient matrix for each band to estimate the correlation between it and adjacent bands [33]. The bands with larger standard deviations and smaller mean values are regarded to be discriminative. It is hypothesized that the redundancy among selected bands depends on the size of coefficient matrix. However, since the spectral curve is usually uneven, the appropriate parameter is difficult to be determined.

### 2.3. Clustering-Based Methods

Clustering-based methods endeavor to divide all bands into different clusters. The most appropriate band (or centroid) in each cluster is selected to constitute final band subset. The Ward’s linkage strategy using mutual information (WaLuMI) and divergence (WaLuDi) are two representative band selection methods [34]. First, a hierarchical clustering based on minimum variance is adopted to partition bands into multiple groups. Next, WaLuMI selects the band with the largest mutual information as the most representative band in its cluster. Similarly, in WaLuDi, the band with highest Kullback–Leibler divergence is regarded as the most representative band.

As a special case of clustering-based methods, there are also studies regarding subspace decomposition, which divides all bands into many sequential subspaces, rather than disordered clusters. For instance, based on the similarity calculated by Euclidean distance, a fast neighborhood grouping band selection method (FNGBS) is proposed [35]. Additionally, only the similarity between bands and cluster center is considered when dividing band subspace to reduce the computational complexity. The optimal clustering framework (OCF) utilizes dynamic programming and decomposes the complex space division into numerous subproblems [36]. The solutions of subproblems can be recombined to solve the initial problem. Compared with ranking-based methods, these methods can reduce the redundancy of obtained bands. Nevertheless, it is still a difficulty for choosing appropriate criteria to evaluate the partitioned clusters.

### 2.4. EA-Based Methods

EA-based methods adopt a population-based strategy to search for approximately optimal band subset. Since the ranking or clustering problems can be regarded as optimization problem, they can be incorporated into EAs. First, EA-based methods generate an initial set of candidate solutions. Next, the genetic operations are implemented to update the population until the convergence condition is reached. The clonal selection algorithm based on maximum information and minimum redundancy (MIMR-CSA) considers the information content and redundancy are considered simultaneously, and proposes the MIMR criterion [37]. The criterion deems that the informative bands are usually redundant due to the high similarity between adjacent bands. In addition, the bands with less information might also provide the supplementary information. Finally, the CSA is adopted to seek an optimal band subset according to the MIMR criterion. The artificial bee colony based on improved subspace decomposition (ISD-ABC) divides the band subspaces according to both the correlation coefficient and spectral curve distribution [38]. Then the ABC is employed to search for informative bands from each subspace by information entropy. However, since the ordered property is neglected and the decomposed band subspaces is always fixed, the redundant bands are easily selected.

## 3. Unsupervised Band Selection Based on Multimodal Evolutionary Algorithm and Subspace Decomposition (MEA-SD)

**Figure 1:**
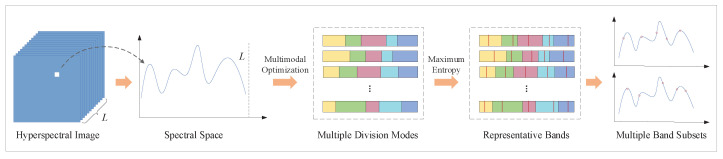
Overall procedure of the proposed method.

Since most of existing methods ignore the diversity of band subsets and the ordered property in hyperspectral bands, a multimodal evolutionary algorithm and subspace decomposition (MEA-SD) method for unsupervised band selection is proposed to alleviate the problems. The overall procedure of the proposed method is shown as Figure 1. Specifically, multimodal optimization is employed to seek out diverse solutions to decompose the spectral space. The band subsets are constituted by selecting the most representative bands from each subspace. In view of the ordered property, the constructed evaluation criterion concentrates on increasing the difference between adjacent subspaces. The procedures of MEA-SD are detailed as follows.

### 3.1. Boundary Encoding Strategy

**Figure 2:**
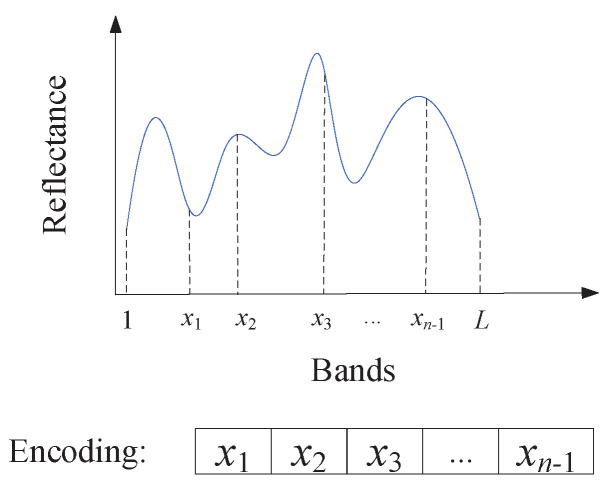
Boundary encoding strategy.

In EA-based methods of band selection, the first step is to encode individuals and generate initial population. Existing encoding methods commonly encode the bands directly. However, adjacent bands may be selected simultaneously due to the high similarity between them [39]. This phenomenon further causes the redundancy of obtained bands. To alleviate this problem, we propose a boundary encoding strategy, which is shown in Figure 2.

In Figure 2, n−1 random bands are selected, and divide the *L*-dimensional band space into *n* subspaces. Correspondingly, the n−1 boundary bands $\left(x_{1},x_{2},\ldots ,x_{n-1}\right)$ are encoded as individuals to construct the initial population in EA. Owing to the encoding, the target of multimodal optimization algorithm is converted to searching for multiple optimal boundary bands. Since the band subset is composed of the most representative bands in each subspace, the adjacent bands are difficult to be selected simultaneously.

### 3.2. Fitness Evaluation Criterion

After encoding boundary bands, the spectral space can be divided into many subspaces. The fitness function is the metric to evaluate the subspaces. The normalized association (NA) is an effective criterion, which is first used to evaluate spectral clustering in [40,41]. The similarity of a cluster *X* can be calculated as Equation (1). Here, *L* is the set of total bands and $\omega_{ij}$ is the similarity between band *i* and *j*. The calculation of $\omega_{ij}$ is shown as Equation (2). Here, $\sigma_{i}=\sqrt{∥x_{i}-x_{d}{∥}^{2}}$, and $x_{d}$ is the dth adjacent band of $x_{i}$ (*d* is set to 7 according to [42]). The clusters with high NA values have a high similarity within groups and low similarity among all groups.

(1) $$N\;A\left(X\right)=\frac{\sum_{i,j\in X}\omega_{ij}}{\sum_{i\in X,j\in L}\omega_{ij}}$$

(2) $$\omega_{ij}=\exp\left(-\frac{∥x_{i}-x_{j}{∥}^{2}}{\sigma_{i}\sigma_{j}}\right)$$

As a special case of band clustering, subspace decomposition divides the band space into sequential subspaces (or clusters). Due to the ordered property and high correlation of contiguous bands, the difference between adjacent clusters is supposed to be more concerned to avoid selecting similar bands. Therefore, the modified version of Equation (1) is used as fitness evaluation criterion, which is formulated as Equation (3). In Equation (3), *K* is the total number of divided subspaces. $X_{adj}$ denotes the adjacent subspaces of $X_{k}$, i.e., only the adjacent subspaces are considered when calculating the similarity between groups rather than all subspaces. By fitness evaluation criterion, the decomposed subspaces are low correlative, and the redundancy of selected bands would be reduced.

(3) $$Fitness\left(X_{k}\right)=\sum_{k=1}^{K}\frac{\sum_{i,j\in X_{k}}\omega_{ij}}{\sum_{i\in X_{k},j\in X_{adj}}\omega_{ij}}$$

### 3.3. Multimodal Optimization Framework

During the search process in MEA, the differential evolution based on fitness Euclidean-distance ratio (FERDE) is adopted as multimodal optimization framework due to its robustness and characteristic without any niche parameters [43]. FERDE consists of three steps, including selection, mutation, and crossover operations. In selection operation, FERDE introduces a novel criterion to evaluate the encoded individual, namely $F\;E\;R_{\left(j,i\right)}$, which is calculated as Equation (4). Here, $p_{i}$ and $p_{j}$ denote the personal best of *i*th and *j*th individual, respectively. $p_{w}$ is the worst-fit solution in the current population. $f(p_{j})$ and $f(p_{w})$ are corresponding fitness values of $p_{j}$ and $p_{w}$. The larger $F\;E\;R_{\left(j,i\right)}$ value indicates that the individual *j* is near-and-better to *i*. Additionally, the roulette selection is employed to select individuals with larger FER values. Then the selected individuals $x_{r1}$, $x_{r2}$ and $x_{r3}$ are used to operate mutation strategy, which is formulated as Equation (5). Here, *D* is the dimension of encoded individuals, namely the number of boundary bands.

(4) $$F\;E\;R_{\left(j,i\right)}=\frac{f\left(p_{j}\right)-f\left(p_{w}\right)}{∥p_{j}-p_{i}∥}$$

(5) $$v_{p}=x_{r1}+rand\left(1,D\right)\cdot \left(x_{r2}-x_{r3}\right)$$

The crossover strategy is next applied to the mutant vector $v_{p}$ and its parent vector $x_{r1}$. It is formulated as Equation (6), where $u_{p,d}$ denotes the *d*th dimension of offspring individual $u_{p}$. CR is the crossover rate, which is the random number between 0 and 1.

(6) $$u_{p,d}=\left\{\begin{array}{cc} v_{p,d}\;\; & \mathrm{if}\;rand_{d}<C\;R\;\mathrm{or}\;d=d_{rand}\\ x_{r1,d} & \mathrm{otherwise} \end{array}\right.$$

By the evolutionary strategies, FERDE converges the solutions towards the individuals with the larger value of FER, respectively. Thus, it can locate the multiple different optima through evolutionary strategies. These obtained solutions represent multiple global or local optimal division modes of spectral space. The detailed steps are shown in Algorithm 1.
**Algorithm 1** Procedure of FERDE.1:**Input:** Hyperspectral image *G*, Size of population *M*, Max iterations $T_{max}$2:**//Initialization**3:Encode and generate initial population P(0) for dataset *G*.4:Calculate its fitness according to Equation (3).5:**//Evolution**6:**while**$t<T_{max}$**do**7:    **for** each individual in P(t) **do**8:        Calculate FER values and select parent individuals.9:        Generate new offspring individuals $u_{p}$ according to Equations (5) and (6).10:       Replace the nearest individual in P(t) if $u_{p}$ is better.11:    **end for**12:    **//Update**13:    P(t)→P(t+1)14:**end while**15:**Output:** Final solution set P(t)

### 3.4. Integrated Utilization Strategy

This section introduces the proposed integrated utilization strategy for the obtained multiple diverse solutions by multimodal evolutionary framework, including representative bands selection and integration operation. For selecting representative bands, the information content in bands can be quantified by information entropy, which is defined as Equation (7). Here, $H_{i}$ is the information entropy of band *i*, Ω is the grayscale color space, and p(z) is the discrete probability distribution of event *z*, which can be calculated by gray histogram method. The band with maximum information entropy is regarded as the most representative band in each subspace.

(7) $$H_{i}=-\sum_{z\in \Omega }p\left(z\right)\log p\left(z\right)$$

**Figure 3:**
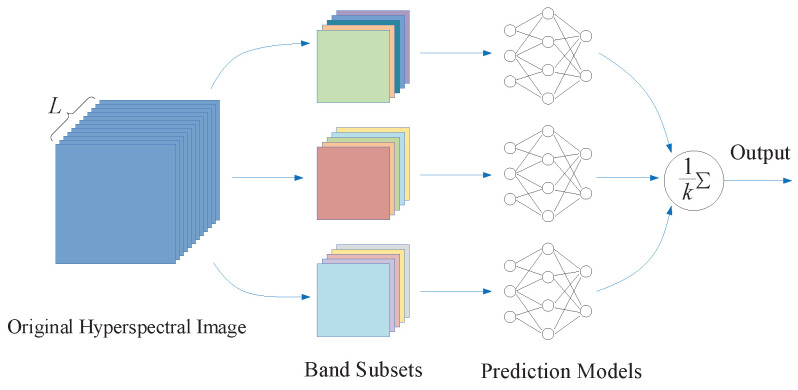
Integration operation using multiple band subsets.

In addition, different from existing band selection methods, the proposed method can obtain several different and optimal (global or local) band subsets within an independent band selection process. These band subsets can express original HSI information from different perspectives. In other words, they may provide much complementary information due to the diversity among these band subsets [37]. The integration operation utilizes multiple band subsets and make a comprehensive prediction, which is shown as Figure 3.

The details of integrated utilization strategy can be described as follows.

- Calculate information entropy of all bands according to Equation (7).
- Sort the obtained solution set *P* according the fitness values, and select the top *k* different individuals, denote as *X*.
- For each individual in *X*, the corresponding band subset is composed of the bands with the maximum entropy in each subspace.
- According to *k* band subsets, operate corresponding pattern recognition tasks (classification or regression), respectively.
- Implement integration operation and output the final prediction results.

In summary, the proposed method adopts the multimodal optimization algorithm and subspace decomposition for selecting appropriate band subsets. Through the designed boundary encoding and modified fitness evaluation criterion, the band space is divided into several sequential subspaces or clusters. Additionally, the informative bands are selected to compose the band subset by the information entropy. Therefore, the obtained band subset is expected to be more discriminative and lower correlative. Another advantage is the utilization of multiple diverse band subsets. By the designed integrated utilization strategy, the final predicted results are more comprehensive.

### 3.5. Computational Complexity

Besides the prediction performance, the computational complexity is also an important evaluation criterion in band selection. In this study, the computational complexity is mainly concentrated on two steps including evolutionary step and representative bands selection step. In evolutionary step, the calculation of FER takes $O(T\;P^{2})$ computational complexity. Calculating similarity matrix in Equation (2) needs $O(N\;L^{2})$ operations. Fitness calculation takes $O(T\;P\;L^{2})$ computational complexity. Here, *T* is the times of actual iterations, *P* is the population size, *N* and *L* are the number of pixels and bands in hyperspectral image. In representative bands selection step, the information entropy calculation needs O(NL) operations. Due to P<T<L≪N, the computational complexity of the proposed method is $O(N\;L^{2})$.

## 4. Experiments on Remote Sensing Datasets

To validate the performance of the proposed method, the experiments are operated on three popular remote sensing datasets, Indian Pines, Pavia University and Salinas in this section. All datasets are from (http://www.ehu.eus/ccwintco).

### 4.1. Description of Remote Sensing Datasets

**Figure 4:**
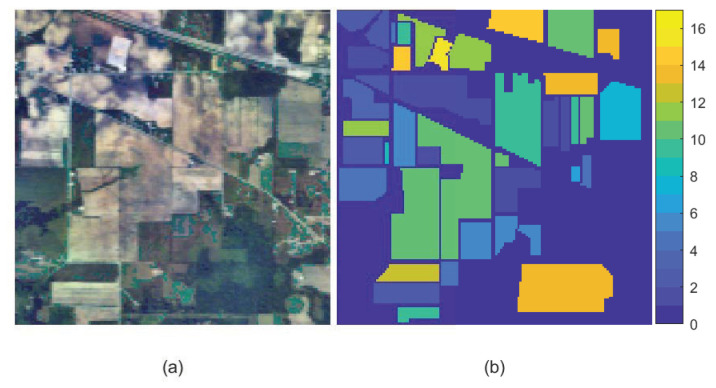
Indian Pines dataset. (**a**) False color image. (**b**) Ground truth.

**Figure 5:**
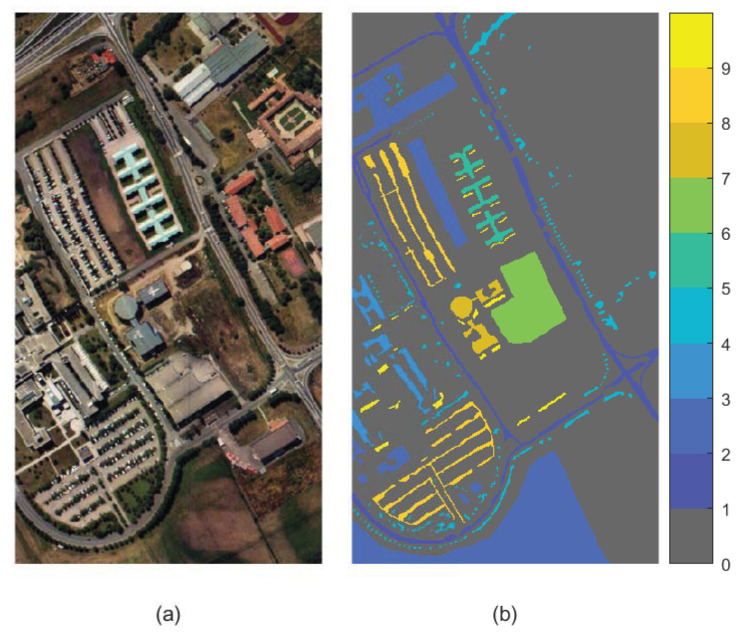
Pavia University dataset. (**a**) False color image. (**b**) Ground truth.

**Figure 6:**
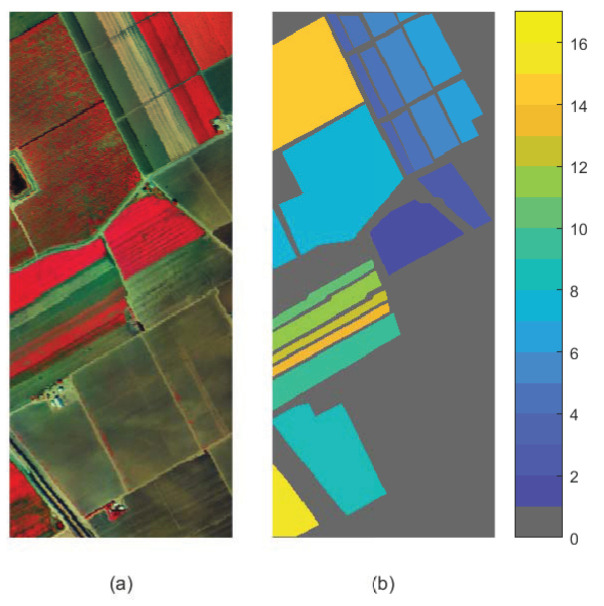
Salinas dataset. (**a**) False color image. (**b**) Ground truth.

(1) Indian Pines: The Indian Pines scene was captured by AVIRIS sensor over the northwestern Indiana. The spectral wavelength ranges from 400 to 2500 nm. It consists of 200 bands (after removing water absorption bands), 145 × 145 pixels and total 16 classes of the land cover objects. Figure 4 shows the false color image (6th, 15th and 23th bands) and ground truth of Indian Pines dataset.

(2) Pavia University: The Pavia University dataset was collected by ROSIS sensor over the Pavia University in Italy. The wavelength is in the range of 430 nm to 860 nm. The dataset has nine classes of ground cover, and 610 × 340 pixels. Overall, 12 noisy bands are discarded, and the remaining 103 bands are used for subsequent experiment. The images of false color (7th, 26th and 46th bands) and ground truth of the dataset are shown in Figure 5.

(3) Salinas: Salinas scene was also obtained over Salinas Valley by AVIRIS in California, USA. Similarly, the wavelength of original hyperspectral image ranges from 400 to 2500 nm. It contains 512 × 217 pixels, and 16 classes of interests. Due to the elimination of 20 water absorption bands, the total 204 bands are used in the experiments. Figure 6 shows the false color image (6th, 15th and 23th bands) and ground truth of Salinas dataset.

The details of these three datasets are described in Table 1.

### 4.2. Experimental Setup

(1) Classification setting: For all band selection methods in experiments, support vector machine (SVM) is adopted as classifier to evaluate the selected band subsets. In SVM, the coefficients of penalty C and gamma are confirmed by five-fold cross validation. In classification, 10% samples randomly chosen from each class are used to train SVM classifiers, the remaining 90% samples are used as testing set to evaluate the classification performance. The proposed method stops its evolution when the maximum iterations reach 300 or the max fitness is not increased in 10 continuous iterations. The population size is set to 50. For reducing instability, all results are calculated and averaged through 30 independent operations.

(2) Comparison algorithms: To verify the superiority of the proposed method, five popular unsupervised band selection methods are adopted, namely E-FDPC [32], WaLuDi [34], TOF [36], MIMR-CSA [37], ISD-ABC [38]. Among the comparisons, E-FDPC is ranking-based unsupervised band selection method. WaLuDi and TOF select the bands using clustering-based method. MIMR-CSA and ISD-ABC are EA-based unsupervised band selection methods. MIMR-CSA employs the clonal selection algorithm to select the bands according to MIMR criterion. ISD-ABC decomposes the subspace through correlation coefficient and spectral characteristics and select bands by ABC. The parameters of maximum iterations and population size of EA-based methods are set as same as the above. All of these used band selection methods have been introduced in Section 2.

### 4.3. Experimental Results

In order to investigate the performance of the proposed method better, the experimental analysis is detailed as the following steps, including parameter analysis, comparisons of experimental results and execution time comparisons.

#### 4.3.1. Parameter Analysis

The proposed method utilizes multiple band subsets to achieve a comprehensive classification performance. The parameter *k* indicates the number of the used band subsets. Therefore, in this section, we first analyze the influences on classification performance with different values of *k*. Specifically, the parameter *k* is set to 1, 3, and 5, respectively. Table 2 describes the corresponding classification results of the proposed method, in which the size of each band subset ranges from 4 to 20. Note that, for *k* = 1, it denotes that only the band subset with the best fitness value is employed. As for *k* = 3 or 5, it indicates that multiple band subsets are used to implement the classification process.

(a) Indian Pines: From Table 2, for *k* = 1, the classification accuracy is significantly improved when the number of used bands changes from 4 to 8. This variation tends to be slow with the continual increasing of the dimension of band subset. As for *k* = 3 or 5, the classification accuracy is further improved. Specifically, compared with results of *k* = 1, the classification accuracy reaches 83.31% and increases by 2.09% when *k* = 3 and the dimension of band subset is 16. The classification accuracy is 83.56% and increases by 2.34% when *k* = 5. It indicates that multiple band subsets can provide complementary information to enhance the classification performance.

(b) Pavia University: Likewise, Table 2 also reveals that the classification accuracy of Pavia University dataset is improved as the increasing of the parameter *k*. Especially, as *k* = 3 and the input dimension is 20, the accuracy is 93.32%. Correspondingly, for *k* = 5, the accuracy improves from 92.08% to 93.56%.

(c) Salinas: For the Salinas dataset, there is also improvement of the classification accuracy when *k* = 3. The classification accuracy reaches 93.51% when the input dimension is 12. When *k* = 5, the proposed method achieves a better classification performance in most of dimensions although the improvement is not obvious compared with *k* = 3.

In summary, the proposed method can seek out multiple different band subsets within a single operation. From Table 2, when single band subset (*k* = 1) is used, the proposed method achieves favorable classification performance. Further, integrate utilization strategy improves the classification accuracy using multiple band subsets, and achieves the best classification performance with *k* = 5. However, the improvement tends to decline when *k* increases from 3 to 5. The reason might be that the redundant band subsets are involved with continued increasing of the parameter *k*. In terms of the number of used band subsets and the time-consuming, *k* = 1 and 3 are preferred and considered in subsequent experiments. Moreover, for *k* = 3, the selected band subsets are listed in Table 3 and Table 4 when the dimensions are 6 and 16, respectively. The results show the difference among obtained band subset. It reveals that the optimal band subsets are not unique.

#### 4.3.2. Comparison of Experimental Results

**Figure 7:**
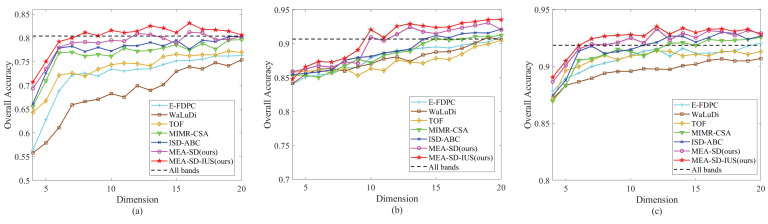
OA curves of different methods on three datasets. (**a**) Indian Pines. (**b**) Pavia University. (**c**) Salinas.

In this subsection, the comparison results of the proposed method and competitors are introduced to demonstrate the superiority of the proposed method. The overall accuracy (OA) and average accuracy (AA) are calculated as evaluation criteria for classification. First, the OA curves of different band selection methods are depicted as Figure 7, in which the dimension of band subset ranges from 4 to 20. Second, more explicitly, Table 5, Table 6 and Table 7 describe the detailed classification results for three HSI datasets when the dimension is 12. Note that, the MEA-SD denotes the proposed method with *k* = 1, i.e., only using single band subset. The MEA-SD-IUS indicates the proposed method with integrate utilization strategy, in which the multiple band subsets are utilized.

(a) Indian Pines: Figure 7a shows the classification results on Indian Pines dataset of proposed methods and competitors. First, the proposed MEA-SD achieves satisfactory classification results using one single band subset. In particular, as the dimension is 16, the accuracy reaches 81.22% and increases by 3.5% compared with ISD-ABC. Then, for the MEA-SD-IUS, the classification accuracies are further improved due to multiple obtained band subset are employed. Through the curves, it is demonstrated that the proposed methods achieve superior classification performance.

(b) Pavia University: The classification results on Pavia University dataset is depicted in Figure 7b. From the figure, when the dimension ranges from 4 to 8, the proposed method surpasses the competitors a little despite the advantage is not obvious. With the increase in the number of bands, the improvement of OA values of five comparing methods tends to be slow. On the contrary, the proposed MEA-SD and MEA-SD-IUS show larger advantage, especially as the size of band subset is from 9 to 14. When the number of bands is greater than 10, the accuracy of proposed method exceeds 90%, and reveals better classification performance.

(c) Salinas: Figure 7c describes the OA curves on Salinas dataset. Similar to the both datasets above, the proposed methods are superior to the competitors in most of the bands. Although the OA values of all methods increase slowly as the number of bands exceeds 7, there is still superior classification performance achieved by proposed methods. When the number of bands is 12, the OA value of proposed MEA-SD reaches 93.30% and increases by 3.5% compared with ISD-ABC.

To sum up, the proposed method achieves superior classification performance compared with five competitors. It can be explained as the following reasons. First, to reduce the correlation of obtained bands, the constructed evaluation criterion for subspace decomposition concentrate more on increasing the difference between neighbor subspaces. The band subset is composed of the bands with maximum information entropy in each subspace. It ensures the band subsets are more discriminative and informative. Second, diverse band subsets acquired by multimodal optimization algorithm are used to improve the classification performance further through integrated utilization strategy.

#### 4.3.3. Comparison of Execution Time

In band selection, the time consumption is also an important indicator. In this subsection, the computational time on the three datasets is presented. All experiments are implemented in MATLAB 2018a on the computer with Intel Core i7-10700 2.9 GHZ CPU and 16 GB RAM. The execution time is calculated and averaged over 30 independent operations. Specifically, the computing time is shown as Table 8 when the number of selected bands is 10. In Table 8, it can be observed that the methods without evolutionary algorithm need less running time in comparison to other competitors. However, the classification performance obtained by these methods are poor. Compared with the other two EA-based methods, MIMR-CSA and ISD-ABC, the proposed method takes less running time and achieves superior classification performance. Therefore, the execution time of the proposed method outperforms the other two EA-based methods.

## 5. Experiments on Mulberry Fruit Dataset

Due to the fast and non-destructive characteristics, the hyperspectral imaging is also applied in the field of chemical components analysis. In this section, the HSI dataset of mulberry fruit is adopted to evaluate the proposed method. The band subsets obtained by the proposed method are used to predict the contents of anthocyanin and flavonoid in mulberry fruits.

The hyperspectral images are collected by HySpex series of HSI spectrometer. The wavelength ranges from 948 nm to 2512 nm. This dataset contains 815 samples and 288 bands with the resolution of 5 nm. The true values of flavonoid and anthocyanin contents are obtained by chromatography method, which is destructive, and needs much time for chemical reagent reaction.

In the experiment, the dimension ranges from 4 to 20, the parameter k is set to 1 and 3. The mean square error (MSE) and $R^{2}$ score between true values and predicted values are calculated to evaluate all the methods. When the $R^{2}$ score is closer to 1, the corresponding method would have a better prediction. The detailed results are shown as follows. Specifically, the selected bands by proposed method on mulberry fruit data set are listed in Table 9 when the numbers of bands are 12 and 18.

**Figure 8:**
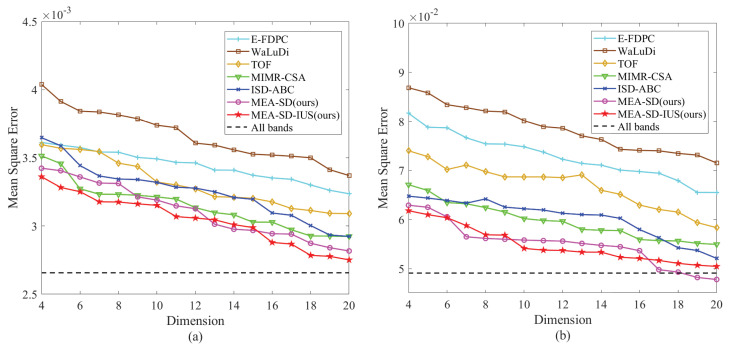
MSE curves of different methods on mulberry fruit dataset. (**a**) Anthocyanin contents. (**b**) Flavonoid contents.

(a) Anthocyanin: For anthocyanin contents in mulberry fruits, Figure 8a shows the predicted MSE values with different dimensions. It can be observed that the overall errors of proposed method and comparisons tend to be reduced. The reason is that the useful band information is provided as the dimension increases. Additionally, among all the methods, the proposed MEA-SD performs well on most of dimension. Specifically, when the dimension is 12, the predicted results obtained by proposed method and comparisons are shown in Table 10. The MSE of MEA-SD is 3.13 × 10${}^{-3}$, and $R^{2}$ score reaches 0.87, which outperform other competitors. Additionally, with the integrated utilization strategy, the MEA-SD-IUS reduces the predicted MSE further.

(b) Flavonoid: The detailed results predicted by different methods are shown in Figure 8b and Table 10. Compared to other methods, EA-based methods achieve better predicted performance. The reason is that evolutionary algorithm usually has better search capability, especially for the higher dimension. Although TOF is a global optimal search method for band selection, its evaluation criterion for band subsets takes all clusters into account. However, in view of the ordered characteristic of bands in hyperspectral image, neighbor bands have stronger similarity. The discrimination between adjacent subspaces should be focused more. From the results, the proposed method achieves satisfactory predicted performance.

## 6. Discussion

Band selection is a combinational optimization task to select informative band subsets and remove redundant bands. In previous studies, many methods neglect the ordered characteristic and easily select adjacent bands, which might have negative influence on hyperspectral image processing. In addition, the optimal band subsets are usually unique, especially for hundreds of hyperspectral bands. The different band subsets could provide supplementary information for each other. However, most of existing studies only find single band subset to represent original hyperspectral image information, and ignore the diversity. In view of these two issue, we propose an unsupervised band selection based on multimodal evolutionary algorithm and subspace decomposition. Compared with previous studies, the first advantage is that the proposed method construct a novel evaluation criterion to increase the difference between adjacent band subspaces. It ensures that the strongly correlative and adajacent bands won’t be chosen simultaneously. The second advantage is that we combine the band selection based on subspace decomposition with multimodal evolutionary algorithm, in which multiple diverse band subsets can be found within an independent operation. Moreover, due to the supplementary information provided by obtained diverse band subsets, this study adopts an integrate utilization strategy to improve the prediction ability further. The performance of the proposed method have been verified by the experiments on three remote sensing data sets and a mulberry fruit dataset. From the results in Figure 7 and Figure 8, it can be seen that the proposed method achieves a higher classification accracy and lower regression error compared with five competitors.

While the proposed method have achieved a superior prediction performance, there are still limitations. The search capability of multimodal evolutionary algorithm for hundreds of spectral dimensions can be improved further [16]. Besides, a self-adaptive strategy to determine the number of clusters or selected bands is worthy of study [28], which is also a challenge in current band selection methods.

## 7. Conclusions

On account of the issues of the redundancy of hyperspectral image and the diversity of band subsets, an unsupervised band selection based on multimodal evolutionary algorithm and subspace decomposition is proposed in this study. First, the subspace decomposition can divide the spectral bands into many sequential subspaces. The band subset is composed of bands with maximum entropy from each band subspace. Therefore, it avoids that the adjacent bands are selected simultaneously. Second, in order to decompose the spectral space better, the multimodal evolutionary algorithm with a constructed evaluation criterion for band subspace is employed. Due to the evaluation criterion, the subspace with larger difference between neighbors is prefered. Besides, the other advantage of multimodal evolutionary algorithm is that it can seek out multiple diverse band subsets which can provide supplementary information. By the integrated utilization strategy, the diverse band subsets can be utilized to improve the prediction performance further. The experiments on Indian Pines, Pavia University, Salinas and mulberry fruit data sets have also demonstrated that the proposed method can achieve a superior performance compared with comparison algorithms.

Nevertheless, there are shortcomings that need to be improved in this study. For instance, how to confirm an appropriate number of selected bands. It is also a difficulty in existing researches of band selection. Besides, the multi-feature fusion, such as morphology and textural features, for hyperspectral image processing is worthy of study. These considerations will be continued in our future work.

## Figures and Tables

**Table 1:** Description of remote sensing datasets.

Dataset	Size	Samples	Classes	Bands
Indian Pines	145 × 145	10,249	16	200
Pavia University	610 × 340	42,776	9	103
Salinas	512 × 217	54,129	16	204

**Table 2:** Classification accuracies (%) of different parameter *k* on three datasets.

Dataset	*k*	4	6	8	10	12	14	16	18	20
Indian Pines	1	69.36	77.99	79.18	79.51	80.94	79.97	81.22	80.70	81.36
	3	70.72	79.23	81.17	81.60	81.43	82.09	83.31	82.72	82.66
	5	71.37	79.89	81.51	81.91	83.20	83.01	83.56	82.98	82.79
Pavia University	1	85.91	86.78	87.31	91.55	91.41	91.78	91.98	92.69	92.08
	3	84.79	87.38	87.85	92.09	92.59	92.65	92.47	93.28	93.32
	5	84.92	87.27	88.70	92.54	92.85	93.18	92.92	93.33	93.56
Salinas	1	88.66	91.54	91.91	92.51	93.30	92.92	93.15	92.74	92.92
	3	89.06	91.84	92.67	92.80	93.51	93.37	93.28	93.09	92.81
	5	89.30	91.59	92.91	92.90	93.30	93.62	93.59	93.51	93.47

**Table 3:** Selected bands by the proposed method on three datasets when the dimension is 6.

Dataset	Selected Bands
	18, 29, 42, 117, 131, 160
Indian Pines	18, 29, 42, 75, 117, 131
	29, 42, 54, 89, 117, 160
	21, 36, 47, 63, 78, 91
Pavia University	11, 21, 40, 63, 78, 91
	21, 36, 49, 63, 77, 91
	15, 34, 45, 57, 120, 164
Salinas	34, 45, 57, 79, 120, 164
	15, 34, 45, 93, 120, 164

**Table 4:** Selected bands by the proposed method on three datasets when the dimension is 16.

Dataset	Selected Bands
	5, 8, 17, 21, 38, 42, 60, 69, 71, 117, 131, 134, 160, 166, 174, 191
Indian Pines	17, 29, 42, 69, 71, 75, 97, 99, 117, 130, 136, 159, 160, 176, 183, 191
	18, 21, 28, 29, 42, 54, 70, 89, 117, 130, 132, 154, 160, 166, 182, 190
	2, 4, 13, 18, 21, 24, 39, 45, 48, 57, 63, 64, 74, 83, 91, 96
Pavia University	2, 15, 18, 21, 23, 34, 40, 49, 55, 59, 63, 67, 71, 78, 83, 91
	4, 13, 21, 31, 36, 38, 41, 47, 57, 58, 63, 65, 76, 87, 91, 103
	8, 15, 21, 45, 52, 72, 88, 93, 94, 120, 125, 135, 158, 164, 187, 191
Salinas	24, 28, 34, 45, 52, 64, 72, 78, 125, 135, 138, 158, 164, 172, 179, 187
	1, 9, 15, 27, 34, 45, 60, 71, 72, 117, 120, 125, 131, 158, 164, 180

**Table 5:** The detailed classification accuracies (%) of different methods on Indian Pines dataset.

Classes	E-FDPC	WaLuDi	TOF	MIMR-CSA	ISD-ABC	MEA-SD (ours)	MEA-SD-IUS (ours)
1. Alfalfa	78.05 ± 7.54	14.63 ± 17.10	65.85 ± 3.93	84.37 ± 9.5	56.10 ± 2.09	85.37 ± 3.55	80.49 ± 2.72
2. Corn-notill	61.13 ± 4.26	63.35 ± 2.05	66.21 ± 1.16	65.86 ± 3.0	62.88 ± 1.28	74.01 ± 2.01	73.46 ± 1.76
3. Corn-mintill	64.79 ± 3.87	57.56 ± 3.83	64.66 ± 0.69	62.45 ± 2.8	63.59 ± 1.24	63.99 ± 0.90	68.61 ± 0.87
4. Corn	48.83 ± 6.45	23.94 ± 3.25	63.38 ± 0.80	73.24 ± 4.1	72.77 ± 2.38	76.06 ± 1.82	77.00 ± 1.13
5. Grass-pasture	87.59 ± 1.71	83.91 ± 1.63	88.51 ± 0.16	86.51 ± 1.5	90.80 ± 0.61	92.13 ± 0.24	90.80 ± 0.17
6. Grass-trees	87.20 ± 0.66	88.45 ± 1.94	87.85 ± 0.17	89.30 ± 0.8	88.58 ± 0.52	91.02 ± 0.48	92.54 ± 0.35
7. Grass-pasture-mowed	76.14 ± 1.81	48.90 ± 1.81	72.49 ± 2.05	84.60 ± 2.7	76.05 ± 3.91	88.41 ± 1.33	88.99 ± 0.76
8. Hay-windrowed	91.86 ± 0.72	97.67 ± 0.92	91.40 ± 0.08	94.98 ± 0.7	92.79 ± 0.49	95.12 ± 0.74	95.58 ± 0.58
9. Oats	51.14 ± 6.23	38.89 ± 15.09	48.60 ± 3.26	62.67 ± 8.3	38.89 ± 4.22	65.35 ± 3.59	74.40 ± 2.23
10. Soybean-notill	74.29 ± 2.40	73.24 ± 3.17	72.11 ± 1.46	76.91 ± 3.2	74.40 ± 1.18	78.39 ± 0.90	79.43 ± 0.53
11. Soybean-mintill	75.02 ± 0.74	72.19 ± 2.08	75.18 ± 0.42	78.05 ± 1.2	81.63 ± 0.57	82.26 ± 0.81	82.81 ± 0.71
12. Soybean-clean	56.93 ± 2.59	42.23 ± 4.28	57.68 ± 1.71	74.29 ± 2.9	79.21 ± 1.80	76.03 ± 1.56	73.78 ± 1.44
13. Wheat	96.22 ± 0.70	88.11 ± 2.76	95.68 ± 0.08	92.97 ± 0.2	97.84 ± 0.43	94.59 ± 0.18	94.62 ± 0.13
14. Woods	85.61 ± 0.92	87.69 ± 1.10	90.68 ± 0.67	92.54 ± 0.9	93.94 ± 0.67	95.00 ± 0.81	96.05 ± 0.47
15. Buildings-grass-trees	44.67 ± 2.28	23.34 ± 2.87	36.60 ± 1.79	36.31 ± 3.3	47.84 ± 2.88	44.96 ± 4.33	41.79 ± 3.67
16. Stone-steel-towers	85.71 ± 3.45	75.24 ± 5.57	80.29 ± 0.41	81.48 ± 1.6	85.95 ± 1.13	89.29 ± 1.84	90.48 ± 1.21
OA	73.44 ± 0.48	69.96 ± 0.71	74.61 ± 0.35	77.21 ± 0.69	78.37 ± 0.67	80.94 ± 0.72	81.43 ± 0.54
AA	72.82 ± 2.90	61.21 ± 4.34	72.32 ± 1.18	77.03 ± 2.92	75.20 ± 1.59	80.75 ± 1.57	81.30 ± 1.17

**Table 6:** The detailed classification accuracies (%) of different methods on Pavia University dataset.

Classes	E-FDPC	WaLuDi	TOF	MIMR-CSA	ISD-ABC	MEA-SD (ours)	MEA-SD-IUS (ours)
1. Asphalt	89.53 ± 0.28	89.65 ± 0.30	91.15 ± 0.14	90.80 ± 0.20	89.18 ± 0.21	92.85 ± 0.23	92.85 ± 0.23
2. Meadows	95.25 ± 0.15	95.85 ± 0.18	96.18 ± 0.20	95.31 ± 0.14	95.98 ± 0.22	96.11 ± 0.17	97.38 ± 0.12
3. Gravel	66.79 ± 1.39	63.68 ± 2.36	65.43 ± 1.16	68.13 ± 1.68	69.75 ± 0.89	74.11 ± 0.73	74.38 ± 0.40
4. Trees	89.51 ± 0.86	87.93 ± 1.51	87.35 ± 0.33	88.87 ± 1.85	89.65 ± 0.36	90.65 ± 0.80	91.88 ± 0.27
5. Painted metal sheets	99.42 ± 0.25	99.39 ± 0.45	99.34 ± 0.30	99.47 ± 0.33	98.59 ± 0.17	99.60 ± 0.12	99.51 ± 0.13
6. Bare soil	73.67 ± 1.48	65.36 ± 3.52	59.36 ± 1.74	67.69 ± 2.2	70.70 ± 0.39	80.20 ± 0.36	82.48 ± 0.35
7. Bitumen	75.52 ± 0.18	74.02 ± 0.69	75.86 ± 0.11	80.86 ± 0.10	71.17 ± 0.25	81.95 ± 0.41	83.54 ± 0.30
8. Self-blocking bricks	88.77 ± 0.29	88.53 ± 0.31	86.63 ± 0.21	87.96 ± 0.55	88.16 ± 0.11	89.20 ± 0.24	91.52 ± 0.21
9. Shadows	96.53 ± 0.20	99.65 ± 0.18	97.65 ± 0.16	99.53 ± 0.23	98.77 ± 0.18	99.55 ± 0.25	99.65 ± 0.15
OA	89.01 ± 0.16	88.05 ± 0.34	87.60 ± 0.16	88.71 ± 0.30	88.91 ± 0.23	91.41 ± 0.17	92.59 ± 0.16
AA	85.40 ± 0.56	83.82 ± 1.06	83.13 ± 0.48	85.71 ± 0.81	84.98 ± 0.31	88.95 ± 0.37	89.97 ± 0.24

**Table 7:** The detailed classification accuracies (%) of different methods on Salinas dataset.

Classes	E-FDPC	WaLuDi	TOF	MIMR-CSA	ISD-ABC	MEA-SD (ours)	MEA-SD-IUS (ours)
1. Brocoli_greenweeds_1	98.67 ± 0.22	98.06 ± 0.14	97.43 ± 0.07	98.45 ± 0.24	98.56 ± 0.18	98.78 ± 0.15	99.45 ± 0.08
2. Brocoli_greenweeds_2	99.73 ± 0.19	99.46 ± 0.12	99.78 ± 0.06	99.69 ± 0.22	99.91 ± 0.07	99.97 ± 0.02	99.94 ± 0.02
3. Fallow	98.71 ± 0.14	98.59 ± 0.17	97.98 ± 0.13	97.47 ± 0.08	98.99 ± 0.24	98.59 ± 0.25	99.33 ± 0.09
4. Fallow_rough_plow	99.44 ± 0.34	99.68 ± 0.23	99.68 ± 0.19	99.60 ± 0.11	99.52 ± 0.35	99.52 ± 0.19	99.44 ± 0.16
5. Fallow_smooth	98.76 ± 0.18	97.76 ± 0.25	98.76 ± 0.10	97.97 ± 0.32	98.55 ± 0.22	98.55 ± 0.18	98.71 ± 0.09
6. Stubble	99.75 ± 0.12	99.64 ± 0.08	99.78 ± 0.05	99.80 ± 0.11	99.83 ± 0.13	99.83 ± 0.09	99.83 ± 0.12
7. Celery	99.38 ± 0.27	99.47 ± 0.14	99.47 ± 0.09	99.25 ± 0.22	99.50 ± 0.13	99.63 ± 0.19	99.60 ± 0.16
8. Grapes_untrained	84.86 ± 0.20	80.39 ± 0.18	84.52 ± 0.12	85.28 ± 0.14	86.18 ± 0.09	88.40 ± 0.15	88.91 ± 0.14
9. Soil_vinyard_develop	98.68 ± 0.13	98.98 ± 0.06	98.92 ± 0.14	99.80 ± 0.07	99.73 ± 0.20	99.91 ± 0.05	99.93 ± 0.05
10. Corn_senesced_green	95.59 ± 0.31	90.20 ± 0.11	93.12 ± 0.08	94.64 ± 0.21	95.02 ± 0.18	95.83 ± 0.12	96.00 ± 0.13
11. Lettuce_romaine_4wk	95.42 ± 0.25	93.76 ± 0.23	95.53 ± 0.15	95.84 ± 0.39	92.92 ± 0.27	98.96 ± 0.14	99.06 ± 0.11
12. Lettuce_romaine_5wk	99.83 ± 0.06	99.88 ± 0.09	99.83 ± 0.05	99.83 ± 0.07	98.87 ± 0.19	99.84 ± 0.05	99.88 ± 0.03
13. Lettuce_romaine_6wk	98.67 ± 0.17	98.42 ± 0.13	98.91 ± 0.20	98.55 ± 0.21	99.03 ± 0.16	99.52 ± 0.25	99.39 ± 0.23
14. Lettuce_romaine_7wk	94.70 ± 0.21	92.63 ± 0.26	95.95 ± 0.12	94.81 ± 0.09	93.67 ± 0.23	97.40 ± 0.27	96.68 ± 0.20
15. Vinyard_untrained	67.59 ± 0.19	65.37 ± 0.15	67.97 ± 0.08	66.54 ± 0.14	69.59 ± 0.17	72.53 ± 0.20	72.86 ± 0.16
16. Vinyard_vertical	97.79 ± 0.26	96.25 ± 0.18	98.59 ± 0.10	98.71 ± 0.21	99.08 ± 0.16	99.04 ± 0.22	99.14 ± 0.19
OA	91.53 ± 0.16	89.79 ± 0.21	91.38 ± 0.08	91.49 ± 0.23	92.13 ± 0.15	93.30 ± 0.19	93.51 ± 0.13
AA	95.07 ± 0.20	93.78 ± 0.16	94.99 ± 0.11	94.96 ± 0.18	95.21 ± 0.19	96.38 ± 0.16	96.49 ± 0.12

**Table 8:** Averaged computing time (s) of different band selection methods.

Data Set	E-FDPC	WaLuDi	TOF	MIMR-CSA	ISD-ABC	MEA-SD (ours)
Indian Pines	0.202	2.238	0.694	4.084	3.253	3.392
Pavia University	0.217	1.449	0.712	2.750	1.898	1.791
Salinas	0.635	2.730	2.127	4.553	5.823	3.542

**Table 9:** Selected bands by the proposed method on mulberry fruit dataset.

The Number of Bands	Selected Bands
	15, 30, 86, 110, 114, 128, 164, 173, 235, 243, 255, 269
12	15, 30, 40, 77, 133, 164, 170, 223, 235, 242, 249, 272
	13, 15, 30, 53, 63, 68, 86, 128, 147, 164, 241, 273
	13, 15, 30, 77, 83, 89, 108, 127, 128, 135, 142, 164, 194, 203, 235, 239, 255, 266
18	2, 30, 72, 110, 127, 128, 135, 144, 148, 170, 194, 198, 235, 241, 251, 258, 267, 279
	2, 15, 30, 63, 76, 93, 110, 128, 142, 146, 164, 172, 229, 235, 245, 250, 270, 277

**Table 10:** Results of MSE and $R^{2}$ score for prediction of Anthocyanin and Flavonoid contents.

	Anthocyanin		Flavonoid	
	MSE ($10^{-3}$)	$R^{2}$	MSE ($10^{-2}$)	$R^{2}$
E-FDPC	3.46 ± 0.18	0.85	7.23 ± 0.82	0.86
WaLuDi	3.61 ± 0.41	0.85	7.86 ± 1.10	0.84
TOF	3.27 ± 0.17	0.86	6.86 ± 0.36	0.86
MIMR-CSA	3.14 ± 0.09	0.87	5.96 ± 0.68	0.88
ISD-ABC	3.28 ± 0.13	0.86	6.13 ± 0.79	0.88
MEA-SD	3.13 ± 0.08	0.87	5.56 ± 0.57	0.89
MEA-SD-IUS	3.06 ± 0.05	0.87	5.37 ± 0.44	0.89

## Data Availability

The remote sensing datasets are from (http://www.ehu.eus/ccwintco). The mulberry dataset forms part of an ongoing study and is available on request from the corresponding author.
